# Kinetics of monocyte subpopulations during experimental cerebral malaria and its resolution in a model of late chloroquine treatment

**DOI:** 10.3389/fcimb.2022.952993

**Published:** 2022-10-14

**Authors:** Jade Royo, Aissata Camara, Benedicte Bertrand, Philippe Batigne, Agnes Coste, Bernard Pipy, Agnes Aubouy, the NeuroCM Group, Dissou Affolabi

**Affiliations:** ^1^ Unité Mixte de Recherche (UMR152) Pharmcochimie et biologie pour le développement (PHARMADEV), Université de Toulouse, French National Research Institue for Sustainable Development (IRD), UPS, Toulouse, France; ^2^ Pharmacy Department, Institut de Recherche et de Développement des Plantes Médicinales et Alimentaires de Guinée (IRDPMAG), Dubréka, Guinea

**Keywords:** cerebral malaria (CM), mice, monocyte, brain, spleen, blood, chloroquine

## Abstract

Cerebral malaria (CM) is one of the most severe forms of malaria and is a neuropathology that can lead to death. Monocytes have been shown to accumulate in the brain microvasculature at the onset of neurological symptoms during CM. Monocytes have a remarkable ability to adapt their function to their microenvironment from pro-inflammatory to resolving activities. This study aimed to describe the behavior of monocyte subpopulations during infection and its resolution. C57BL/6 mice were infected with the *Plasmodium berghei* ANKA strain and treated or not with chloroquine (CQ) on the first day of the onset of neurological symptoms (day 6) for 4 days and followed until day 12 to mimic neuroinflammation and its resolution during experimental CM. Ly6C monocyte subpopulations were identified by flow cytometry of cells from the spleen, peripheral blood, and brain and then quantified and characterized at different time points. In the brain, the Ly6C^int^ and Ly6C^low^ monocytes were associated with neuroinflammation, while Ly6C^hi^ and Ly6C^int^ were mobilized from the peripheral blood to the brain for resolution. During neuroinflammation, CD36 and CD163 were both involved *via* splenic monocytes, whereas our results suggest that the low CD36 expression in the brain during the neuroinflammation phase was due to degradation. The resolution phase was characterized by increased expressions of CD36 and CD163 in blood Ly6C^low^ monocytes, a higher expression of CD36 in the microglia, and restored high expression levels of CD163 in Ly6C^hi^ monocytes localized in the brain. Thus, our results suggest that increasing the expressions of CD36 and CD163 specifically in the brain during the neuroinflammatory phase contributes to its resolution.

## Introduction

Malaria remains the deadliest parasitic disease worldwide, with 627,000 deaths reported in 2020 (WHO, 2021). Cerebral malaria (CM) is one of the most severe states of malaria and is a neuropathology that may lead to death or neurological sequelae. Despite a decline in malaria mortality since the use of artemisinin derivatives, the case fatality rates related to CM in African children remain in the range of 10%–40% (Dondorp et al., 2010; Royo et al., 2019; Conroy et al., 2021). The mechanisms involved in the pathophysiology of CM are still incompletely understood, although three mechanisms are thought to be crucial. Firstly, the sequestration of *Plasmodium falciparum*-infected red blood cells (iRBCs) in the cerebral microvasculature, demonstrated through postmortem studies (Pongponratn et al., 2003; Taylor et al., 2004; Milner et al., 2015), causes microvascular obstruction, ischemia, and hypoxia (Dondorp et al., 2004; Penet et al., 2005). Secondly, the anticoagulant and cytoprotective functions of the protein C-containing system are dysregulated due to the specific interaction between the parasite protein PfEMP1 and the endothelial receptor EPCR (Petersen et al., 2015). Thirdly, systemic inflammation due to malaria infection combined with activation of the brain’s vascular endothelium leads to increased circulating levels of pro-inflammatory cytokines and chemokines, increased adhesion receptor expression on the endothelium, increased iRBC sequestration, and increased recruitment of leukocytes (Medana and Turner, 2006; Wykes and Good, 2009; Keswani et al., 2016; Gowda and Wu, 2018). These phenomena create a local inflammatory and oxidative environment that ultimately leads to blood−brain barrier (BBB) disruption and central nervous system damage (Becker et al., 2004; Pais and Penha-Gonçalves, 2018).

The experimental murine model of cerebral malaria (ECM) has highlighted the central role of CD8 T cells in ECM development through their implication in endothelial cell apoptosis (Nitcheu et al., 2003; Howland et al., 2015). The implication of monocytes/macrophages in the pathophysiology of CM has also been evidenced both in humans (Pongponratn et al., 2003; Stanisic et al., 2014) and in a murine model, where these cells have been shown to accumulate in the brain microvasculature at the onset of neurological symptoms (Niewold et al., 2018; Sierro and Grau, 2019). As demonstrated in ECM, monocytes are primarily associated with pathogenesis, are a major source of pro-inflammatory cytokines during CM (Grau et al., 1987), and are implicated in CD8 T-lymphocyte accumulation in the brain through chemokine production. Indeed, the depletion of monocytes/macrophages with clodronate liposome injection 5 days post-infection was shown to result in the recruitment of natural killer (NK) cells and CD4^+^ and CD8^+^ T cells to the brain (Pai et al., 2014). However, monocytes are known for their capacity to rapidly mobilize to the inflammation sites and for their wide range of functions from pro-inflammatory to resolving activities (Guilliams et al., 2018).

Monocytes are a heterogeneous population comprising three subpopulations according to their expression levels of CD14 [a lipopolysaccharide (LPS) co-receptor] and CD16 (low-affinity Fcγ receptor III) in humans and according to their Ly6C expression in mice. Although the distribution of these three subsets among mononuclear cells is very different between humans and mice (classical CD14^+^CD16^−^, 80%–95%; intermediate CD14^+^CD16^+^, 2%–11%; and non-classical CD14^low^CD16^+^, 2%–8% *versus* Ly6C^high^, 40%–45%; Ly6C^int^, 5%–32%; and Ly6C^low^, 26%–50%, respectively), their general functions are similar (Yang et al., 2014). While all three subpopulations are capable of phagocytosis, CD14^+^CD16^−^/Ly6C^hi^ and CD14^+^CD16^+^/Ly6C^int^ monocytes are described as pro-inflammatory and reactive oxygen species (ROS)-producing, CD14^+^CD16^+^/Ly6C^int^ monocytes are known for their antigen-presenting ability, and CD14^low^CD16^+^/Ly6C^low^ monocytes are characterized by their tissue repair activity and their role in the surveillance of BBB integrity (Ginhoux and Jung, 2014). However, depending on the pathological context, the role of the subpopulations may be considered favorable or deleterious. For example, during experimental autoimmune encephalomyelitis and dextran sulfate sodium (DSS)-induced colitis, the infiltration of Ly6C^hi^ monocytes is thought to be responsible for the subsequent pathology through their pro-inflammatory activity (Ajami et al., 2011; Zigmond et al., 2012). Conversely, the ability of Ly6C^int^ to produce nitric oxide (NO) and tumor necrosis factor (TNF) has been identified as necessary for the elimination of several pathogens (*Toxoplasma gondii*, *Trypanosoma brucei*, *Leishmania major*, and *Listeria monocytogenes*) (reviewed in Guilliams et al., 2018).

With respect to CM, a few studies have focused on the role of monocyte subpopulations in pathology. In Beninese children presenting with CM, we previously demonstrated an increased risk of death in children displaying a lower percentage of CD14^low^CD16^+^ monocytes, suggesting a role of this subpopulation in achieving better clinical outcomes (Royo et al., 2019). Pai et al., using *Plasmodium berghei* ANKA (PbA)-infected C57BL/6 mice, found that Ly6C^hi^ monocytes were the main brain-sequestered leukocyte population, with CD8 T cells appearing at the stage of neurological symptoms and interacting with the vascular endothelium. At the same time, in blood circulation, they noticed the disappearance of Ly6C^low^ monocytes (Pai et al., 2014). Using a specific *t*-distributed stochastic neighbor embedding analysis of their flow cytometry data, Niewold et al. demonstrated the accumulation of Ly6C^low^ monocytes in the brain on day 8 (D8) post-infection in PbA-infected CBAJ mice. Early administration of immune-modifying particles specifically targeting Ly6C^low^ monocytes improved survival by 50%, suggesting a detrimental role of this subpopulation during ECM (Niewold et al., 2018). Taken together, these results prompted us to explore the precise kinetics of monocyte subpopulations during ECM in the spleen, blood, and brain and their modulation through the expression of membrane receptors.

## Materials and methods

### Experimental animals and ethical considerations

Six-week-old male and female C57BL/6 mice weighing 19–22 g were obtained from Janvier Laboratory (Toulouse, France). Mice were maintained under standard and constant laboratory conditions (unlimited access to food and tap water, adapted enrichment, 23°C–25°C, relative humidity of approximately 60%, and 12/12-h light/dark cycles). All animal experiments were conducted with respect to animal welfare and were approved by the Midi-Pyrénées Ethics Committee for animal experiments in Toulouse (France) under permit number APAFIS#5921-2016070118008477v3.

### Experimental model of the study

**Figure 1:**
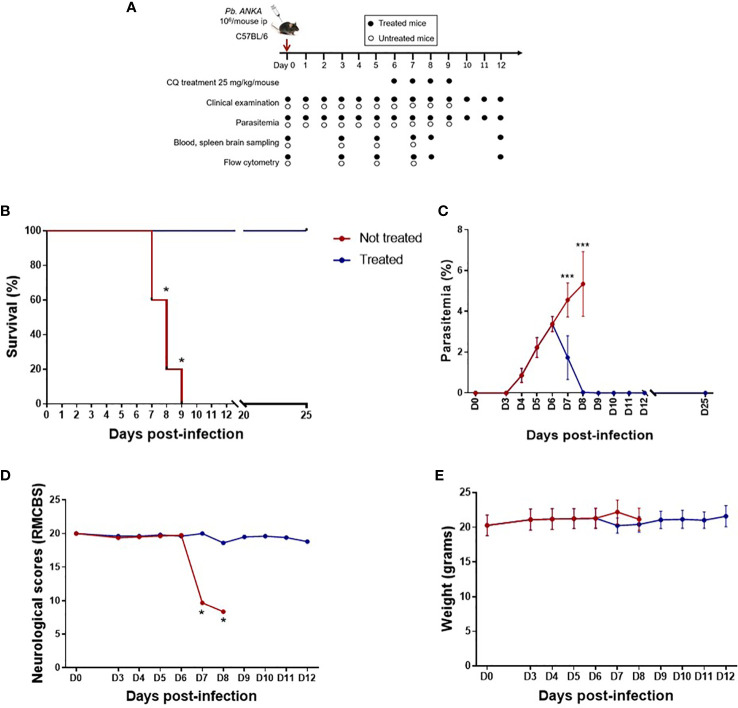
Late treatment with chloroquine (CQ) prolongs survival and limits parasitemia and neurological damage. **(A)** *Plasmodium berghei* ANKA-infected C57BL/6 mice treated intraperitoneally from day 6 (D6) to D9 post-infection with 25 mg kg^−1^ day^−1^ CQ and followed by clinical and parasitological examination, organ sampling, and flow cytometry analysis. **(B)** Survival curve in treated and untreated mice. **(C)** Course of parasitemia in treated and untreated mice. **(D)** Rapid murine cerebral behavior scale (RMCBS) scores during the course of infection in treated and untreated mice. **(E)** Weight of treated and untreated mice. Treated and untreated mice were compared for all parameters on D7, D8, and D9 using the Mann–Whitney *U* test. *p < 0.05, ***p < 0.0005.

The rodent malaria parasite *P. berghei* strain ANKA (kindly given by A. Berry, INFINITY Research Unit, Toulouse, France) was used throughout the study. **Figure 1A** shows the experimental model used in this study. Mice were intraperitoneally infected with 1.10^6^ P*. berghei* ANKA parasites suspended in 200 µl of 0.9% NaCl on D0 and divided into two groups: treated and untreated. In the untreated group, mice were followed during the course of infection, and groups of three to five mice were sequentially sacrificed on D0, D3, D5, and D7. The second group was treated with chloroquine (CQ) on D6, D7, D8, and D9 (intraperitoneal injection of 25 mg/kg of CQ per day per mouse in 400 µl of 0.9% NaCl) and subsequently sacrificed on D7 (called D7T), D8 (D8T), and D12 (D12T). Weight, parasitemia, and neurological symptoms were monitored daily, while survival was monitored twice daily. Parasitemia was monitored from D3 by microscopic counting of May–Grünwald–Giemsa-stained blood smears (RAL 555 kit, RAL Diagnostics, Martillac, France) and determined as follows: [(number of iRBC)/(total number of RBC counted)] × 100.

### Rapid murine coma and behavioral tests

To evaluate brain damage, the rapid murine cerebral behavior scale (RMCBS) was employed daily according to a previously described methodology (Carroll et al., 2010). Ten parameters were evaluated: gait, balance, exploratory behavior, grooming, body position, tactile escape reflex, ear pavilion reflex, limb strength, toe reflex, and aggressiveness. Each parameter was scored from 0 to 2, with a score of 2 indicating the highest function.

### Collection of spleen, brain, and peripheral blood from mice

The spleens and brains were collected immediately after death at the different time points, mechanically homogenized in 7 ml of phosphate-buffered saline (PBS) and filtered through a 100- or 40-µm nylon cell strainer. The remaining brain and spleen pellets were centrifuged (400 × *g*, 10 min). For the brain, the mononuclear cells were separated from the digested tissue using density gradient centrifugation (800 × *g*, 20 min) (Percoll^®^, Sigma, St. Louis, MO, USA) and then washed and resuspended in PBS. For the spleen, RBCs in the cell pellet were lysed by adding 1 ml of ammonium–chloride–potassium (ACK) buffer and centrifuged (400 × *g*, 10 min). The cell pellets were finally suspended in 1 ml of PBS. Peripheral blood was obtained by cardiac puncture in heparinized syringes and centrifuged (400 × *g*, 5 min) to separate plasma. Peripheral blood mononuclear cells (PBMCs) were isolated from the pellet by density gradient centrifugation (Lymphoprep, STEMCELL Technologies, Saint-Egrève, France) and washed in PBS.

### Flow cytometry

Mononuclear cells from the brain, splenocytes, and PBMCs were stained with fluorescein-labeled monoclonal antibodies (mAbs) specific for cell surface markers, as follows: F4/80-PeVio615, CD45-PeVio770, CD11b-FITC, Ly6C-VioBlue451, Ly6G-APC, MHC-II-PerCP-Vio700 (Miltenyi Biotech, Bergisch Gladbach, Germany), CD36-PE (Santa Cruz Biotechnology, Santa Cruz, CA, USA), and CD163-AF680 (www.antibodies-online.com). A Zombie Aqua Fixable Viability Kit (BioLegend, San Diego, CA, USA) was used to exclude dead cells. The labeling protocol was performed following the manufacturer’s guidelines. Unlabeled cells, bead and cell monolayers, and isotype controls were also used as controls. Acquisition was performed on an LSR Fortessa cytometer (BD Biosciences, Franklin Lakes, NJ, USA), and data were analyzed using FlowJo™ software.

### Statistical analysis

All data are expressed as the mean ± standard deviation (SD) and were analyzed using GraphPad Prism software (version 9.3.0). Parasitemia, survival, the neurological scores, and weight were compared between treated and untreated mice using the Mann−Whitney *U* test. For analyses of the population percentages and the mean fluorescence intensity (MFI), a one-way ANOVA was performed before two-by-two comparisons using unpaired two-tailed Student’s *t*-test. Significant differences were defined as *p* < 0.05.

## Results

### Treatment with chloroquine from D6 to D9 prevents neurological damage and death

To mimic human CM and its resolution, C57BL/6 mice were infected with the *P. berghei* ANKA strain and treated with or without CQ from D6 to D9 (**Figure 1A**). As shown in **Figure 1B**, untreated mice died between D7 and D9. Before treatment at D6, the mean parasitemia was approximately 3.4% (**Figure 1C**), and parasitemia increased to 5.3% in untreated mice. In the treated group, CQ treatment resulted in a rapid decrease in parasitemia and an increase in mouse survival (**Figures 1B, C** **)**. With respect to the neurological score (RMCBS) assessed using 10 behavioral parameters, a maximum score of 20 was obtained in all mice until D6, suggesting little or no visible neurological symptoms (**Figure 1D**). In contrast, the CQ-treated mice maintained a stable neurological score, with the score decreasing by half and becoming less than 10 in untreated mice by D7, highlighting significant neurological damage at this stage of infection and validating the CQ administration protocol from D6 (**Figure 1D**). The weight of the infected mice did not change during malaria infection or after treatment (mean weight in grams ± SD for all weights from D0 to D12: 21.0 ± 0.162 and 21.1 ± 0.217 in treated and untreated mice, respectively) (**Figure 1E**). Here, we postulate that this model mimics neuroinflammation on D7 in untreated mice, but mimics the resolution of inflammation by D12. In treated mice, D7 and D8 reflected the effect of CQ treatment. Indeed, the reported half elimination time for CQ was 1.5 h in Swiss mice infected with *Plasmodium chabaudi* at 3.5% parasitemia treated with 5 mg/kg CQ (Cambie et al., 1994). Thus, the half elimination time of CQ in mice presenting a similar parasitemia and treated with 25 mg/kg CQ may be 7.5 h.

### Ly6C^int^ and Ly6C^low^ monocytes are associated with neuroinflammation in the blood and brain, while Ly6C^hi^ and Ly6C^int^ are mobilized from peripheral blood to the brain for resolution

To better understand the involvement of monocyte subpopulations during CM and its resolution, the spleen, blood, and brain of *P. berghei* ANKA-infected mice were collected at different time points after malaria infection and resolution (D0, D3, D5, D7, D8, and D12). The brains of non-perfused mice were collected to evaluate the complete kinetics of the monocytes present in the brain, including those adherent to the vascular endothelium. Monocyte kinetics and the levels of expression of surface markers were compared between all compartments.

**Figure 2:**
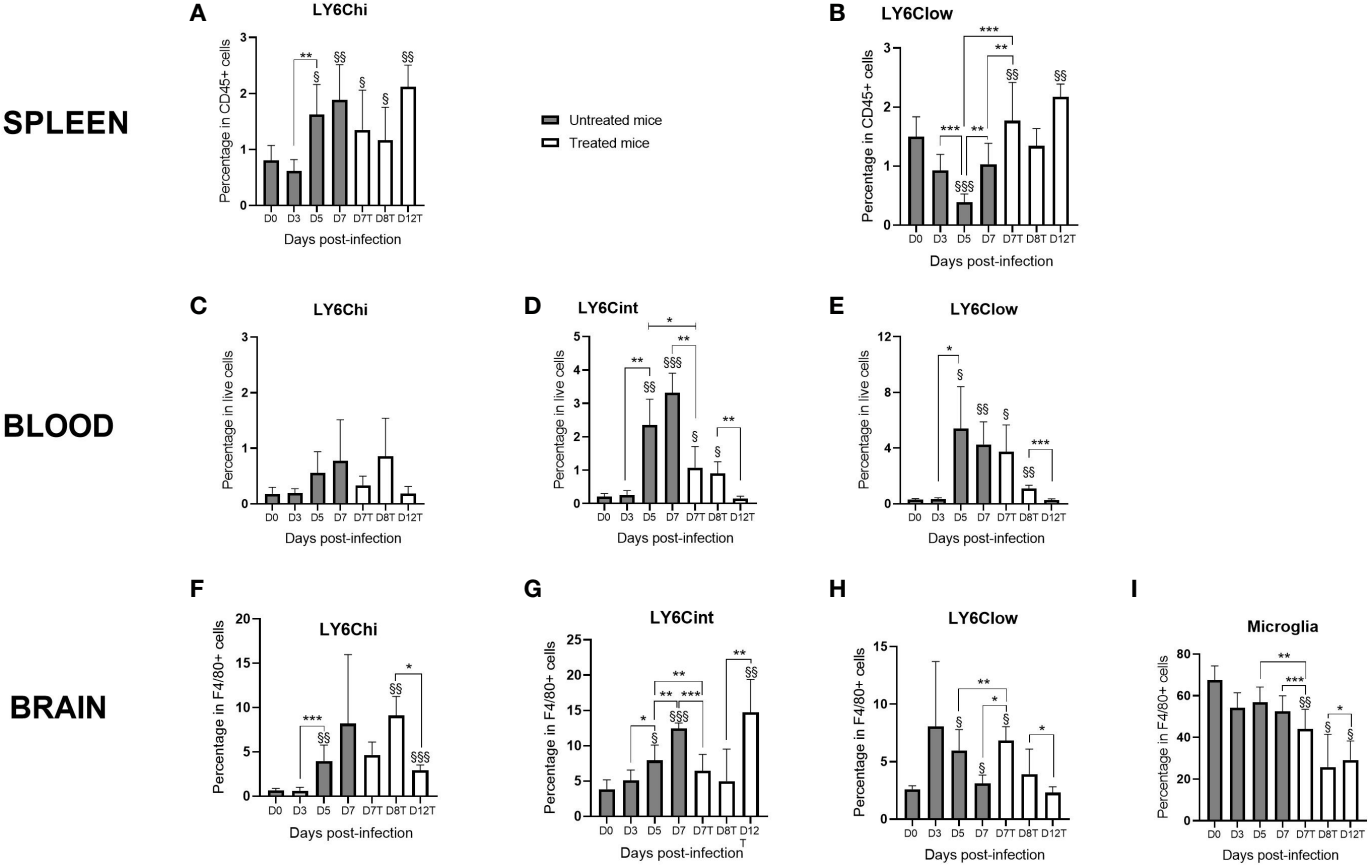
Monocyte subpopulations evolve differently in the spleen, peripheral blood, and brain during infection and resolution in response to chloroquine (CQ) treatment. The spleen, blood, and the brain were sampled at different time points of infection with the *Plasmodium berghei* ANKA strain in CQ-treated and untreated mice. Monocyte subpopulations, as well as the microglia, were identified and quantified from live cells by flow cytometry using FlowJo. **(A, B)** Percentages of Ly6C^hi^ **(A)** and Ly6C^low^ **(B)** monocytes in the spleen in treated and untreated mice. **(C–H)** Three percentages of the monocyte subpopulations in the peripheral blood **(C–E)** and in the brain **(F–H)** during kinetics in treated and untreated mice. **(I)** Percentage of the microglia during kinetics. The results are presented as *curves* and *bars* for better visualization of the kinetics and the significant differences. For each subpopulation, one-way ANOVA first confirmed that the means were not equal before two-by-two comparisons using an unpaired two-tailed Students *t*-test. Significant differences between consecutive time points are shown with an *asterisk*, while differences from day 0 (D0) are shown with a *section symbol*. *One symbol* denotes *p* < 0.05, *two symbols p* < 0.005, and *three symbols p* < 0.0005.

Monocyte subpopulations and the microglia were analyzed using flow cytometry (see the gating strategy in **Supplementary Figure S1**). **Figure 2** shows the kinetics of the monocyte subpopulations among live cells present in the spleen, peripheral blood, and brain of infected mice. In the spleen, the percentage of Ly6C^hi^ monocytes increased from D0 to D7 with or without treatment (**Figure 2A**). Ly6C^low^ monocytes increased from D5 to D7, and CQ treatment exacerbated this increase (**Figure 2A**). From D7 until D12, the percentages of both Ly6C^hi^ and Ly6C^low^ monocytes remained high in treated mice compared to those at D0. For the monocytes present in peripheral blood and in the brain, we observed relatively similar kinetics for Ly6C^hi^, Ly6C^int^, and Ly6C^low^, except at D12, the time of CM resolution (**Figure 2B**). In both compartments, the percentages of Ly6C^int^ and Ly6C^low^ monocytes increased between D0 and D7 in untreated mice (**Figure 2B**), implicating both subpopulations in neuroinflammation. In the brain, levels of the microglia decreased from D5 to D7 and D8 upon CQ treatment, followed by a slight increase on D12 (**Figure 2C**). On D12, the percentages of all three monocyte subpopulations in the blood were low (**Figure 2B**), whereas Ly6C^hi^ and Ly6C^int^ monocytes in the brain increased to levels above those at baseline (**Figure 2C**), suggesting the mobilization of these cell populations for inflammatory resolution from the peripheral blood to the brain. With respect to Ly6C^low^ monocytes present in the brain, basal percentages were restored on D12 (**Figure 2C**).

Interestingly, we observed that the effect of treatment by D7 was different among the three compartments: in the spleen and the brain, the treatment resulted in increased Ly6C^low^ subpopulations (compared to those on D7 and D5 in untreated mice) (**Figures 2A, C** **)**. For the microglia and Ly6C^int^ monocytes present in the blood and brain, we observed a decrease in these subpopulations after treatment (**Figures 2B, C** **)**. In the peripheral blood, no treatment effect was observed in the Ly6C^hi^ or the Ly6C^low^ subpopulation (**Figure 2B**). **Supplementary Figure S2** shows the percentages of the subpopulations among monocytes. In **Supplementary Figures S2A, B**, it is obvious that, in the spleen and the peripheral blood, the percentages of Ly6C^hi^ and Ly6C^low^ monocytes were inversely correlated, which reinforces the idea that Ly6C^low^ monocytes are derived from Ly6C^hi^ monocytes (Niewold et al., 2018). Interestingly, Ly6C^low^ monocytes replaced Ly6C^hi^ in the spleen from D5 (**Supplementary Figure S2A**) and in the blood from D3 (**Supplementary Figure S2B**). In the brain, this observation applied to Ly6C^hi^ and Ly6C^int^ monocytes from D5 (**Supplementary Figure S2C**).

### CD36 and CD163 are involved in neuroinflammation *via* splenic monocytes and *via* the microglia and Ly6C^hi^ monocytes in the brain during the resolution phase

**Figure 3:**
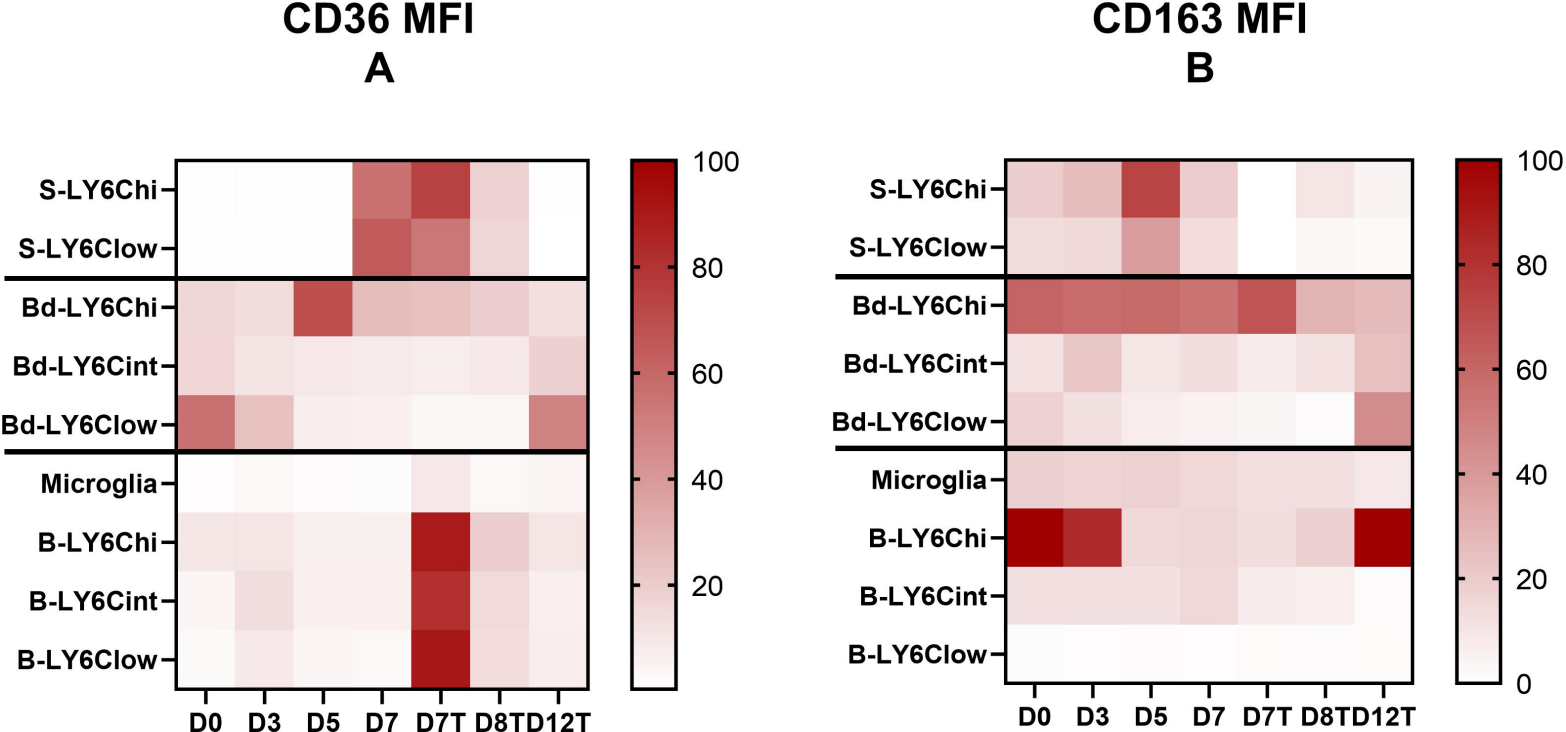
CD36 and CD163 receptors are differentially expressed in terms of expression level and evolution during the kinetics of infection and its resolution by the monocyte subpopulations from one compartment to another. **(A, B)** Mean fluorescence intensity (MFI) of CD36 **(A)** and CD163 **(B)** by the different Ly6C monocyte subpopulations and the microglia characterized in the spleen, blood, and brain were analyzed using FlowJo during *Plasmodium berghei* ANKA infection in chloroquine (CQ)-treated and untreated mice. To obtain comparable data during kinetics between the different subpopulations of each compartment studied (i.e., the spleen, blood, brain), the heatmap presents the data as a ratio to the highest MFI value obtained during the kinetics ×100 for each compartment.

To characterize the monocyte subpopulations during the course of malaria infection and treatment, the expression levels of CD36 and CD163 receptors were assessed using flow cytometry (**Figure 3** and **Supplementary Figure S3**). **Figure 3** displays the ratios of the highest value of the three, or four for the brain, subpopulations calculated to avoid strong differences in the expression levels between organs that do not allow visualization of marker expression differences during kinetics. **Supplementary Figure S3** presents the true values of marker expression and their significant differences. CD36 and CD163 are two scavenger receptors involved in the elimination of iRBCs through direct phagocytosis and hemoglobin/haptoglobin complexes, respectively (Tenhunen et al., 1968; McGilvray et al., 2000). CD163 contributes to the anti-inflammatory response and is associated with M2 macrophage polarization, whereas CD36 is implicated in glycosylphosphatidylinositol (GPI) recognition by monocytes through its role as a co-receptor of TLR2, resulting in TNF production (Erdman et al., 2009).

Interestingly, receptor expressions varied among compartments. For CD36, we noted much lower expression levels by monocytes found in the peripheral blood than by monocytes isolated from the spleen and brain (**Supplementary Figures S3A, C, E**). Increased expression of CD36 was observed in the spleen on D7 before treatment at the peak of infection and inflammation, whereas in the brain, high expression levels were observed in monocyte subpopulations after treatment on D7 (**Figure 3A** and **Supplementary Figures S3A, E**). In the blood, CD36 was more strongly expressed by Ly6C^hi^ monocytes on D5 and by Ly6C^low^ monocytes on D0 and D12 (**Figure 3A** and **Supplementary Figure S3C**). These results suggest the involvement of CD36 expressed by Ly6C^hi^ and Ly6C^low^ monocytes of the spleen and Ly6C^hi^ monocytes of the blood in GPI recognition and/or the elimination of iRBCs through phagocytosis on D7 and D5, respectively. In the brain, on D7, we observed strikingly higher expression levels of CD36 in treated mice compared to untreated mice in all subpopulations (**Figure 3A** and **Supplementary Figure S3E**). This difference may be attributed to CQ treatment (Liang et al., 2004). However, the lack of difference between the treated and untreated groups on D7 in the other two compartments may not support this hypothesis or may suggest a brain-specific mechanism. In addition, in the brain, an increased expression of CD36 by the microglia and by Ly6C^int^ and Ly6C^low^ monocytes was observed before treatment on D3 (**Figure 3A** and **Supplementary Figure S3E**), which may reflect the activity of non-opsonic phagocytosis of these populations at this stage of infection or their implication in GPI signaling and the pro-inflammatory response. The latter hypothesis is more likely, as parasitemia on D3 was still close to zero (**Figure 1B**). Finally, we observed a significant increase in the expression of CD36 by the microglia on D12 compared to D8 (*p* = 0.036) and D0 (*p* = 0.0095) in treated mice (**Figure 3A** and **Supplementary Figure S3E**). This result suggests a role of this receptor in inflammatory resolution through the microglia.

Concerning CD163, an increase in its expression was observed on D5 compared to D3 in both monocyte subpopulations found in the spleen (**Figure 3B** and **Supplementary Figure S3B**), suggesting increased removal of hemoglobin/haptoglobin complexes at this point during infection. In the peripheral blood, Ly6C^hi^ monocytes highly expressed this receptor until D7 in both treated and untreated mice, with the expression levels subsequently decreasing by half from D8 in treated mice (**Supplementary Figure S3D**). Ly6C^int^ and Ly6C^low^ monocytes expressed CD163 much more weakly than did Ly6C^hi^ monocytes in the peripheral blood, but there was an increase in CD163 expression by these two subpopulations on D12 (**Figure 3B** and **Supplementary Figure S3D**), suggesting a contribution of both monocyte subpopulations to resolution *via* this receptor. In the brain, CD163 was almost uniformly expressed by the microglia and Ly6C^int^ during infection, with a decrease from D7 posttreatment, while Ly6C^low^ monocytes exhibited very low expression levels of this receptor (**Figure 3B** and **Supplementary Figure S3F**). Interestingly, CD163 was strongly expressed by Ly6C^hi^ monocytes in the brain before infection and until D3, repressed between D5 and in treated mice until D8, and finally restored on D12 (**Figure 3B** and **Supplementary Figure S3F**). This result suggests strong polarization of this cell population into inflammatory monocytes from D5 to D7 and until D8 in treated mice, followed by M2 polarization by D12 during inflammatory resolution.

**Figure 4:**
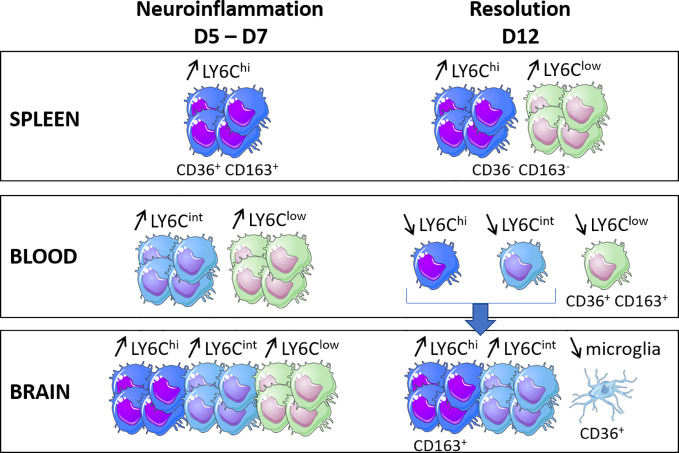
Schematic illustration of the main results obtained for the evolution of the percentages of the monocyte subpopulations in the three compartments (i.e., the spleen, blood and brain) and their CD36 and CD163 receptor expression phenotypes during the two major phases of the infection kinetics (neuroinflammation and its resolution) in a model of experimental cerebral malaria.

**Figure 4** summarizes the main results obtained for the evolution of the percentages of monocyte subpopulations in the three compartments examined (the spleen, blood, and brain) and their CD36 and CD163 receptor expression phenotypes during the two major phases of infection kinetics, neuroinflammation and its resolution.

## Discussion

CM is a particularly problematic disease in sub-Saharan Africa. Despite the administration of effective antimalarial drugs, it unfortunately leads to very high mortality rates of approximately 30%, as reported in our last study conducted in Benin (Brisset et al., 2022). This pathology is also associated with neurological sequelae in patients who survive (WHO, 2014). Although the late management of these cases likely contributes to the severity of the disease, it appears that impairment of the cellular immune response also contributes to fatal outcomes and/or neurological sequelae due to BBB damage (Ghazanfari et al., 2018). Because monocytes are key cells in the modulation of immunity and their role during CM is still controversial, we focused our study on monocyte behavior in a murine model of ECM and its resolution.

Our data suggest that Ly6C^low^ monocytes are derived from Ly6C^hi^ monocytes, especially in the spleen and blood, where the percentages of these subpopulations changed in inverse proportions. In the brain, our results indicate that Ly6C^hi^ monocytes differentiate mainly into Ly6C^int^ monocytes. Monocytes originate from the bone marrow and enter the bloodstream as Ly6C^hi^ CCR2^+^ monocytes, where they can differentiate into Ly6C^low^ monocytes (Yona et al., 2013; Ginhoux and Jung, 2014; Yang et al., 2014). Ly6C^int^, or Ly6C^middle^, are thought to be an intermediate population between Ly6C^hi^ and Ly6C^low^, so this subpopulation is less documented. However, in humans, their CD14^+^ CD16^+^ cell counterpart has been reported to increase in many noninfectious diseases, such as coronary arterial disease (Kashiwagi et al., 2010), atherosclerosis (Wildgruber et al., 2009), hemophagocytic syndrome (Takeyama et al., 2007), and cancer (Tanaka et al., 1999). In our study, the Ly6C^int^ and Ly6C^low^ monocytes detected in the blood and the brain were also increased at the peak of inflammation compared to those on D0. Ly6C^int^ monocytes are inflammatory cells that exhibit similar properties to Ly6C^hi^ monocytes, known for their quick recruitment to inflammatory sites, their high phagocytic capacity, and their ability to secrete ROS and inflammatory cytokines (Yang et al., 2014). Conversely, Ly6C^low^ monocytes are described as anti-inflammatory, promoting M2 polarization and IL-10 production, patrolling the endothelial surface of blood vessels to coordinate its repair if necessary (Auffray et al., 2007).

Interestingly, CD36 and CD163, the two membrane receptors studied here, were expressed very differently in terms of level and evolution during kinetics by the subpopulations from one compartment to another, suggesting a specific phenotype associated with the localization of each monocyte subpopulation. On D7, the Ly6C^hi^ and Ly6C^low^ cell subpopulations were characterized by low expressions of CD36 in the blood and brain compared to the levels observed in the brains of treated mice at this point of infection. The expression of CD36 was indeed elevated to similar levels in the three monocyte subpopulations in the brain on D7 in treated mice. CQ has been shown to severely affect endolysosomal system degradation (Mauthe et al., 2018) and was previously shown to increase the protein expression of CD36 in macrophages (Liang et al., 2004), suggesting that CD36 is undergoing degradation in the brain of the untreated mice in our study. Interestingly, CD36 was overexpressed by the microglia on D12, the time of resolution in this model, compared to D8 in treated mice, at a much higher level than that on D0. CD36 was also highly expressed on D12 by Ly6C^low^ monocytes from the peripheral blood (**Figure 4**). These results suggest that increasing the expression of CD36 in the brain would be beneficial, as already proposed by others (Patel et al., 2007; Olagnier et al., 2011; Ren, 2012). Interestingly, the role of CD36 in the resolution of neuroinflammation in different noninfectious models has been previously reported, such as in Alzheimer’s disease, stroke, ischemic brain due to middle cerebral artery occlusion, brain hematoma, or germinal matrix hemorrhage (Ballesteros et al., 2014; Woo et al., 2016; Zhang et al., 2018; Zhao et al., 2020; Davaanyam et al., 2021; Dobri et al., 2021). In a murine model of Alzheimer’s disease, the microglial expression of CD36 was downregulated, while the pharmacological activation of CD36 was associated with increased Aβ clearance (Dobri et al., 2021). In stroke and ischemia models, increased expressions of CD36 were demonstrated mainly by microglial cells in a PPARγ-dependent manner, which was associated with the resolution of neuroinflammation through phagocytosis (Ballesteros et al., 2014; Zhao et al., 2020). In our study, the observed overexpression may be associated with the removal of blood cell debris and the clearance of parasites. However, Woo et al. concluded that the cell surface CD36 expressed in the post-ischemic brain originates from the peripheral blood rather than from the microglia. This was not the case in this ECM model, for which the increased CD36 expression by the microglia was observed on D12. In the brain, the effect of CQ in this model was striking, showing a specific effect on the brain compared to the other two compartments studied, the peripheral blood and the spleen (**Figure 4**). Thus, the brain-specific immunomodulatory properties of CQ, and in particular its ability to inhibit the degradation of CD36, constitute an avenue to explore in the context of CM.

At the peak of neuroinflammation, splenic Ly6C^hi^ and Ly6C^low^ monocytes also highly expressed CD36 and CD163 in untreated mice (**Figure 4**). The spleen constitutes a key organ for iRBC elimination through highly phagocytic red pulp macrophages and antigen-presenting dendritic cells (Del Portillo et al., 2012). These cells originate from CCR2^+^ Ly6C^hi^ monocytes produced and egressed from the bone marrow to the peripheral blood before invading the tissues *via* the bloodstream (Sponaas et al., 2009; Ginhoux and Jung, 2014). Splenic Ly6C^hi^ monocytes have been shown to display phagocytic properties and the capacity for ROS production, two mechanisms suggested to explain their ability to control parasite development in *P. chabaudi*-infected mice (Sponaas et al., 2009). In our study, the high expression of CD36 by this subpopulation is in line with the high phagocytic activity in the spleen, although this activity was not sufficient to control the infection in untreated mice. Somewhat surprisingly, Ly6C^low^ monocytes also expressed CD36 at high levels similar to those of Ly6C^hi^. In humans, CD14^low^ CD16^+^ cells, the Ly6C^low^ monocyte counterpart, have been reported to be poor phagocytic cells with a low CD36 expression (Wong et al., 2011; Aubouy et al., 2015; Royo et al., 2019). However, during chronic infection with *T. gondii*, Ly6C^low^ monocytes have been shown to highly express CD36, similar to Ly6C^hi^ monocytes, combined with the increased phagocytic capacity of β-amyloid (Möhle et al., 2016), suggesting that this subpopulation may display phagocytic activity during infection settings.

Both cellular subpopulations also highly expressed CD163 on D5 in the spleen, which is when parasitemia increases (**Figure 4**). CD163 is known for its capacity to clear free hemoglobin under conditions of high hemoglobin release resulting from hemolysis, an important step to restoring homeostasis. This hemoglobin uptake activates HO-1, which catabolizes hemoglobin to control the release of heme through hemoglobin degradation to Fe(II), carbon monoxide (CO), and biliverbin (Abraham and Drummond, 2006). In our study, the high expression of CD163 by splenic monocytes was likely a counterregulatory mechanism against the inflammation resulting from heme release related to high parasite multiplication. In contrast, our data also revealed a drastic decrease in CD163 expression by the Ly6C^hi^ monocytes present in the brain from D3 to D5 and D7 in untreated mice, with restoration of the expression levels on D12 in treated mice (**Figure 4**). This lower expression in the brain reflects an M1 phenotype associated with the critical neuroinflammatory phase of the infection. It was previously demonstrated that the neuroinflammation observed during ECM could be resolved by the pharmacological induction of HO-1 or treatment with CO in mice (Pamplona et al., 2007). Our results suggest that CD163, the starting point of the CD163/HO-1 axis, could also constitute a pharmacological target in the brain at the peak of neuroinflammation to help its resolution. To test this hypothesis, the protective role of Ly6C^hi^ CD163^+^ monocytes could be evaluated by transferring these cells into untreated mice before neuroinflammation.

## Collaborators, NeuroCM Group

Dissou Affolabi Pediatric Department, Calavi Hospital, Calavi, Benin; Jules Alao Paediatric Department, Mother and Child University and Hospital Center CHU-MEL, Cotonou, Benin; Daniel Ajzenberg Tropical Neuroepidemiology, INSERM UMR 1094, Limoges, France; Linda Ayédadjou Paediatric Department, Mother and Child University and Hospital Center CHU-MEL, Cotonou, Benin; Gwladys Bertin MERIT, Sorbonne Paris Cité, IRD, 75006, Paris, France; Bibiane Biokou Pediatric Deparstment, Mother and Child University and Hospital Center CHUMEL, Cotonou, Benin; Farid Boumédiène Tropical Neuroepidemiology, INSERM UMR 1094, Limoges, France; Josselin Brisset Infectious diseases and tropical medicine department, Limoges University Hospital, Limoges, France; Michel Cot MERIT, Sorbonne Paris Cité, IRD, 75006, Paris, France; Jean-Eudes Degbelo Institut de Recherche Clinique du Bénin - IRCB, Calavi, Benin; Philippe Deloron MERIT, Sorbonne Paris Cité, IRD, Paris, 75006, France; Ida Dossou-Dagba Paediatric Department, Calavi Hospital, Calavi, Benin; Latifou Dramane Laboratoire de Parasitologie-Mycologie, AP-HP, Hôpital Bichat, Paris; Jean François Faucher EpiMaCT, INSERM, IRD, Université de Limoges, Limoges, France; Sandrine Houzé Parasitology Laboratory, Hopital Bichat - Claude-Bernard, APHP, Paris, and French Malaria Reference Center, Hôpital Bichat, APHP, Paris, France; Sayeh Jafari-Guemouri MERIT, Sorbonne Paris Cité, IRD, Paris, 75006, France; Claire Kamaliddin MERIT, Sorbonne Paris Cité, IRD, Paris, 75006, France; Elisée Kinkpé Paediatric Department, Calavi Hospital, Calavi, Benin; Anaïs Labrunie NET, INSERM, Université de Limoges, Limoges, France; Yélé Ladipo Pediatric Department, Mother and Child University and Hospital Center CHUMEL, Cotonou, Benin; Thomas Lathiere Ophtalmology department, Limoges University Hospital, Limoges, France; Achille Massougbodji Institut de Recherche Clinique du Bénin IRCB, Calavi, Benin; Audrey Mowendabeka Paediatric Department, Hopital de la Mère et de l’Enfant, Limoges, France; Jade Papin MERIT, Sorbonne Paris Cité, IRD, Paris, 75006, France; Pierre-Marie Preux EpiMaCT, INSERM, IRD, Université de Limoges, Limoges, France; Marie Raymondeau NET, INSERM, Université de Limoges, Limoges, France; Darius Sossou Laboratoire de Parasitologie-Mycologie, AP-HP, Hôpital Bichat, Paris; Brigitte Techer MERIT, Sorbonne Paris Cité, IRD, Paris, 75006, France; Bertin Vianou UMR152 PHARMADEV, Université de Toulouse, IRD, UPS, France; Laurence Watier Center for Research in Epidemiology and Population Health CESP, INSERM U1018, Paris-Saclay University, UVSQ, Montigny-Le-Bretonneux, France.

## Data availability statement

The original contributions presented in the study are included in the article/**Supplementary Material**. Further inquiries can be directed to the corresponding author.

## Ethics statement

This study was reviewed and approved by the Midi-Pyrénées Ethics Committee for animal experiments in Toulouse (France).

## Author contributions

AA and BP conceived and designed the project. JR performed all the experiments and the data analysis. AiC performed the behavior tests on mice and helped BB and AgC with the collection of the spleen, brain, and peripheral blood until the cell counts. PB managed the handling of the mice and ensured that it was carried out according to the ethical rules. AA and JR wrote the manuscript. All authors reviewed the manuscript before submission. All authors contributed to the article and approved the submitted version.

## Funding

This work was funded by the French National Research Agency (ANR-17-CE17-0001). In addition to the lab experiments, this grant financed the doctoral fellowship of JR.

## Acknowledgments

We want to thank Alexia Zakaroff-Girard and Elodie Riant from the cytometry technical platform (Rangueil Hospital, Toulouse, France) for their excellent skills and their great and kind help in flow cytometry.

## Conflict of interest

The authors declare that the research was conducted in the absence of any commercial or financial relationships that could be construed as a potential conflict of interest.

## Publisher’s note

All claims expressed in this article are solely those of the authors and do not necessarily represent those of their affiliated organizations, or those of the publisher, the editors and the reviewers. Any product that may be evaluated in this article, or claim that may be made by its manufacturer, is not guaranteed or endorsed by the publisher.

## Supplementary material

Supplementary Figure 1 Gating strategy for monocyte subpopulations in the spleen, blood and brain. The cells of interest were first identified by the SSC and FSC criteria before exclusion of doublets and dead cells using Live Dead labelling (see methods for details). **(A)** For the spleen, CD45^+^ cells were selected for the identification of Ly6C^hi^ and Ly6C^low^ monocyte subpopulations among Ly6C^+^ CD11b^+^ cells. **(B)** For blood, the three Ly6C monocyte subpopulations were identified among Ly6C^+^ CD11b^+^ cells. **(C)** For the brain, F4/80^+^ cells were first selected to identify microglia and myeloid cells in CD45^+^ CD11b^+^ cells and monocyte subpopulations among myeloid cells *via* Ly6C labelling.

Supplementary Figure 2 Evolution of the percentages of Ly6C monocyte subpopulations among monocytes in three compartments (spleen, blood, brain) in *P. berghei* ANKA-infected mice treated or not treated with chloroquine (CQ). The spleen, blood and brain were sampled at different time points of *P. berghei* ANKA infection in CQ-treated and untreated mice, and monocyte subpopulations were identified and quantified among monocytes by flow cytometry on FlowJo. **(A)** Percentages of Ly6C^hi^ and Ly6C^low^ monocytes in the spleen. Percentages of Ly6C^hi^, Ly6C^int^ and Ly6C^low^ monocytes in blood **(B)** and in the brain **(C)** in treated and untreated mice.

Supplementary Figure 3 Expression of CD36 and CD163 in Ly6C monocyte subpopulations and in microglia evolved significantly in the spleen, blood and brain during the kinetics of *P. berghei* ANKA infection and its resolution in chloroquine-treated and untreated mice. Mean fluorescence intensity (MFI) of CD36 and CD163 by the different Ly6C monocyte subpopulations and by microglia characterized in the spleen, blood and brain were analyzed in FlowJo. Data are presented as raw MFI values. For each subpopulation, a one-way ANOVA first confirmed that means were not equal before two-by-two comparisons using an unpaired two-tailed t test. **(A)** CD36 and **(B)** CD163 MFI values of Ly6C monocyte subpopulations present in the spleen. **(C)** CD36 and **(D)** CD163 MFI values of Ly6C monocyte subpopulations present in the peripheral blood. **(E)** CD36 and **(F)** CD163 MFI values of Ly6C monocyte subpopulations and microglia present in the brain. Significant differences between consecutive time points are shown with ^*^, while differences to D0 are shown with ^§^. One symbol means P < 0.05, two symbols P < 0.005 and 3 symbols P < 0.0005.
