# Supplementary figures and images for: Kinetics of monocyte subpopulations during experimental cerebral malaria and its resolution in a model of late chloroquine treatment
Source: Front Cell Infect Microbiol. 2022 Oct 14;12:952993. doi: 10.3389/fcimb.2022.952993 (PMC9614070; doi:10.3389/fcimb.2022.952993)

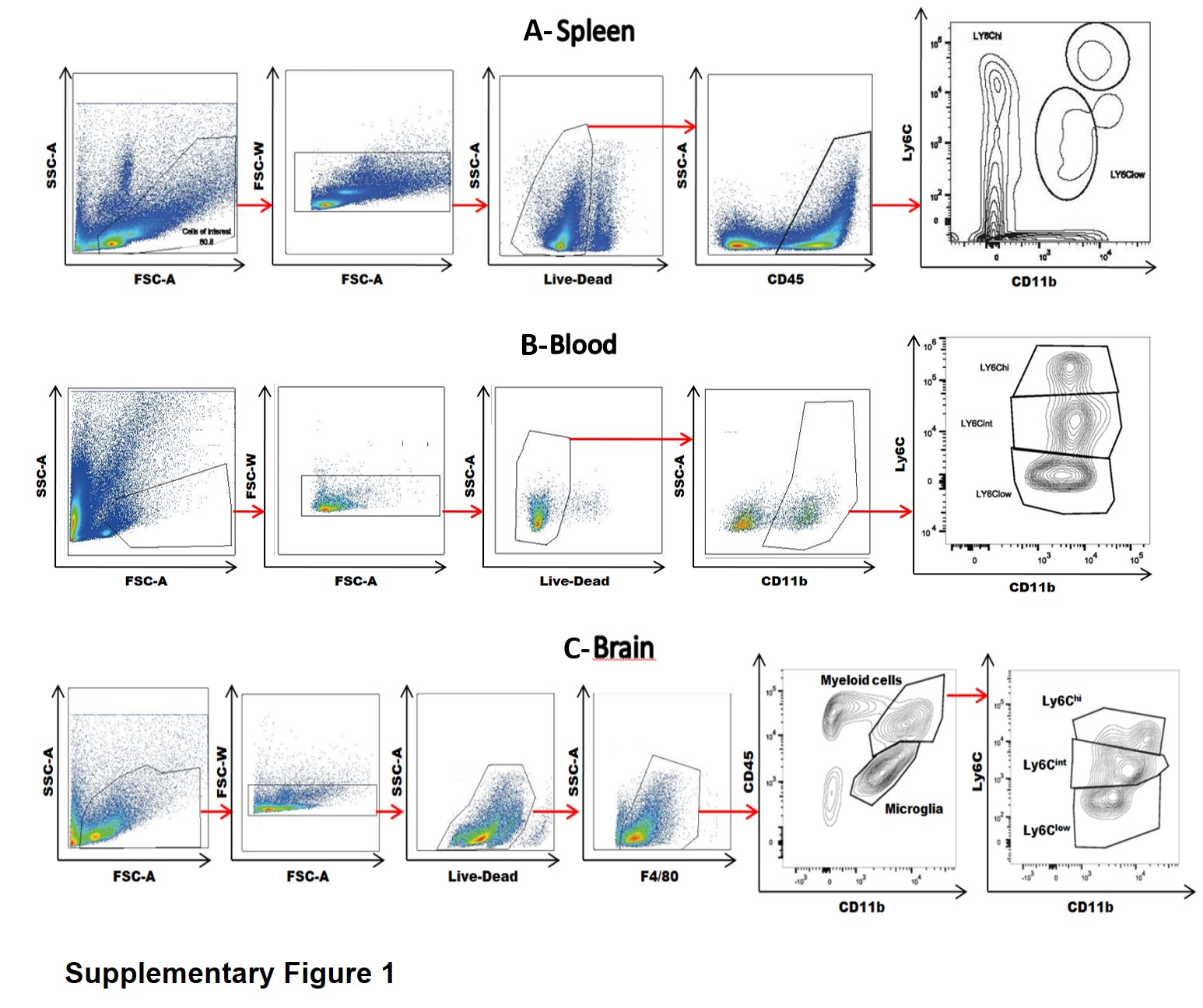

Supplement: Supplementary Figure 1 — Gating strategy for monocyte subpopulations in the spleen, blood and brain. The cells of interest were first identified by the SSC and FSC criteria before exclusion of doublets and dead cells using Live Dead labelling (see methods for details). (A) For the spleen, CD45+ cells were selected for the identification of Ly6Chi and Ly6Clow monocyte subpopulations among Ly6C+ CD11b+ cells. (B) For blood, the three Ly6C monocyte subpopulations were identified among Ly6C+ CD11b+ cells. (C) For the brain, F4/80+ cells were first selected to identify microglia and myeloid cells in CD45+ CD11b+ cells and monocyte subpopulations among myeloid cells via Ly6C labelling. [file Image_1.jpeg]

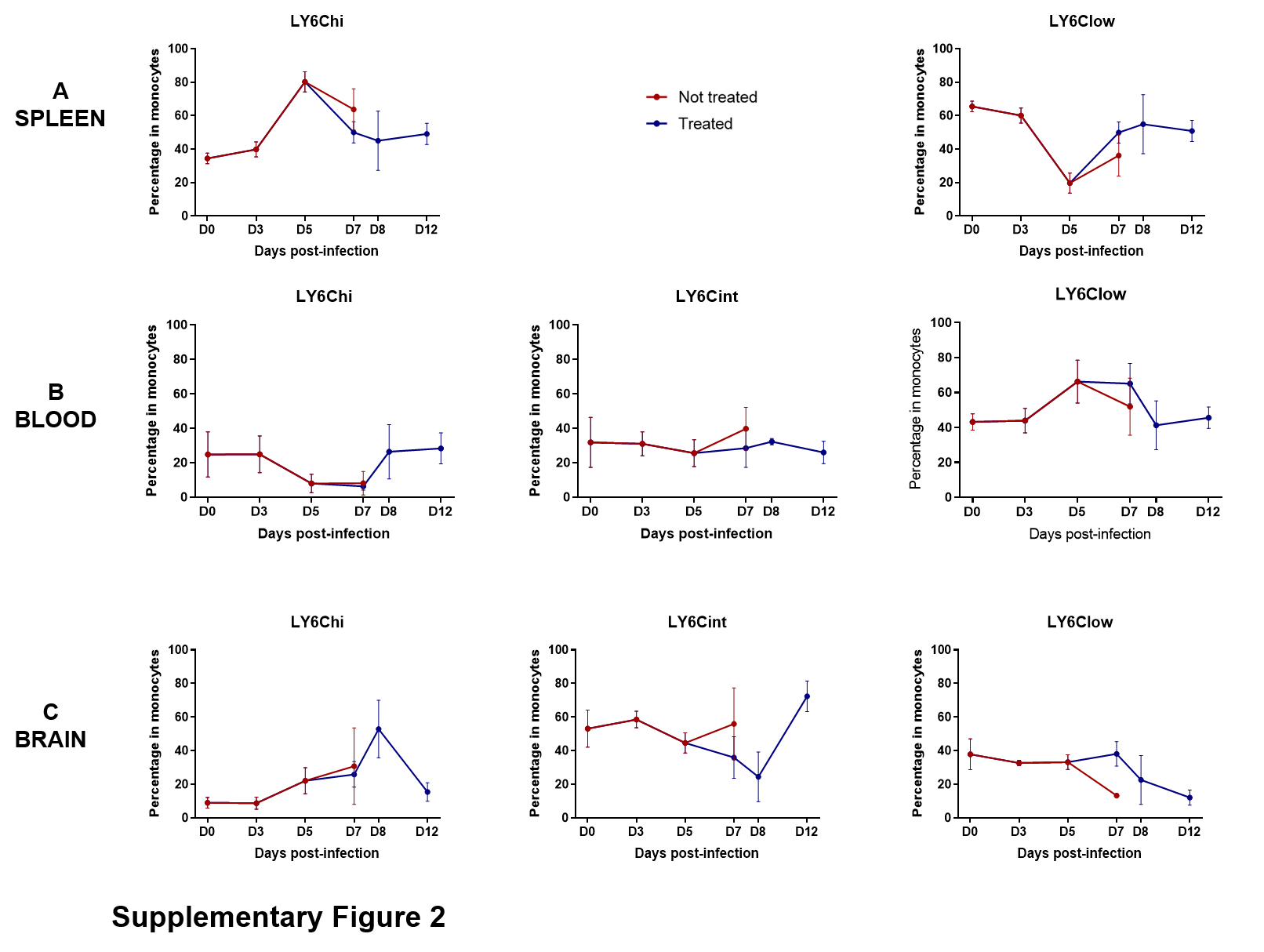

Supplement: Supplementary Figure 2 — Evolution of the percentages of Ly6C monocyte subpopulations among monocytes in three compartments (spleen, blood, brain) in P. berghei ANKA-infected mice treated or not treated with chloroquine (CQ). The spleen, blood and brain were sampled at different time points of P. berghei ANKA infection in CQ-treated and untreated mice, and monocyte subpopulations were identified and quantified among monocytes by flow cytometry on FlowJo. (A) Percentages of Ly6Chi and Ly6Clow monocytes in the spleen. Percentages of Ly6Chi, Ly6Cint and Ly6Clow monocytes in blood (B) and in the brain (C) in treated and untreated mice. [file Image_2.jpeg]

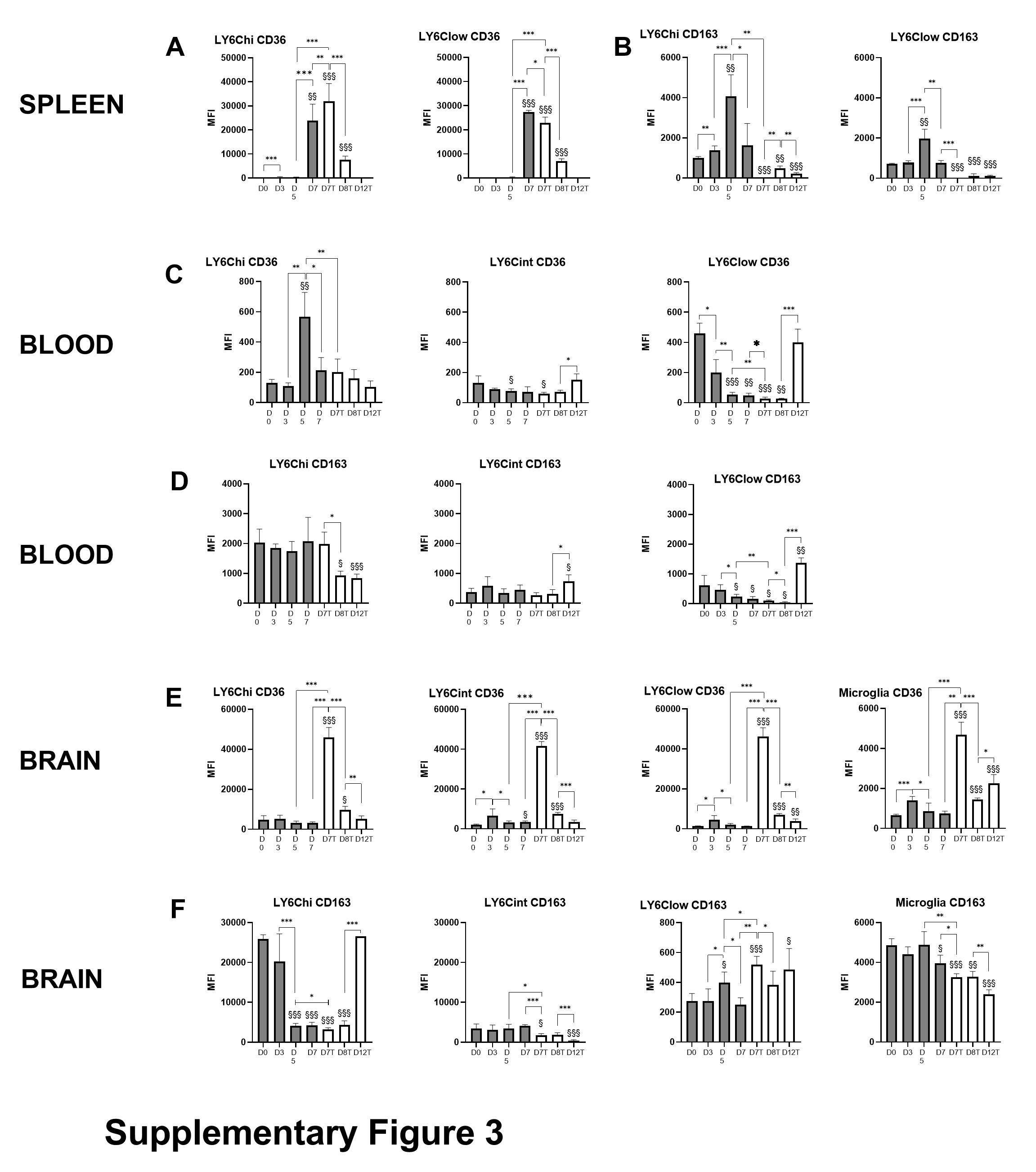

Supplement: Supplementary Figure 3 — Expression of CD36 and CD163 in Ly6C monocyte subpopulations and in microglia evolved significantly in the spleen, blood and brain during the kinetics of P. berghei ANKA infection and its resolution in chloroquine-treated and untreated mice. Mean fluorescence intensity (MFI) of CD36 and CD163 by the different Ly6C monocyte subpopulations and by microglia characterized in the spleen, blood and brain were analyzed in FlowJo. Data are presented as raw MFI values. For each subpopulation, a one-way ANOVA first confirmed that means were not equal before two-by-two comparisons using an unpaired two-tailed t test. (A) CD36 and (B) CD163 MFI values of Ly6C monocyte subpopulations present in the spleen. (C) CD36 and (D) CD163 MFI values of Ly6C monocyte subpopulations present in the peripheral blood. (E) CD36 and (F) CD163 MFI values of Ly6C monocyte subpopulations and microglia present in the brain. Significant differences between consecutive time points are shown with *, while differences to D0 are shown with §. One symbol means P < 0.05, two symbols P < 0.005 and 3 symbols P < 0.0005. [file Image_3.jpeg]
